# A Randomized, Double-Blind, Controlled Clinical Study on the Curative Effect of Huaier on Mild-to-Moderate Psoriasis and an Experimental Study on the Proliferation of Hacat Cells

**DOI:** 10.1155/2018/2372895

**Published:** 2018-08-29

**Authors:** Dongqiang Su, Xuening Zhang, Likun Zhang, Jin Zhou, Feng Zhang

**Affiliations:** ^1^Department of Dermatology, The First Affiliated Hospital, Harbin Medical University, Harbin 150001, China; ^2^Department of Dermatology, The Third Affiliated Hospital, Qiqihar Medical College, Qiqihar 161000, China; ^3^Department of Hematology, The First Affiliated Hospital, Harbin Medical University, Harbin 150001, China

## Abstract

The antitumor effects of Huaier have been recently revealed. However, no research has been conducted on the effects of Huaier on keratinocyte proliferation and for the treatment of psoriasis. Hacat cells were treated with different concentrations of Huaier for different periods of times. The effects on cell proliferation and vitality and on the cell cycle were detected. Patients with mild-to-moderate psoriasis were randomized and divided into two groups in a double-blind manner. The experimental group was given sugar-free Yinxie granules and Huaiqihuang (HQH) granules, and the control group was given sugar-free Yinxie granules and placebo. After 4 weeks, various therapeutic indexes were compared. Huaier significantly inhibited Hacat cell proliferation, suppressed vitality, and blocked the cell cycle in the G1 phase compared with the control group (P < 0.01, respectively). After treatment for 4 weeks, the number of patients between the two groups that experienced a 50% reduction in the Psoriasis Area and Severity Index (PASI 50), PASI 75 and PASI 90, was significantly different (P <0.01). The body surface area (BSA) affected by psoriasis and static physician's global assessment (sPGA) was significantly reduced (P < 0.01); additionally, a significant improvement in the Dermatology Life Quality Index (DLQI) (P < 0.01) was observed. Huaier has shown promising effects in both clinical and experimental setting in this preliminary study and it might provide some benefit in the treatment of psoriasis vulgaris in the future.

## 1. Introduction

Psoriasis is a chronic and debilitating immune-mediated inflammatory skin disease that affects 1%–3% of the global population [1]. Approximately 80% of individuals with psoriasis have a mild-to-moderate form of the disease [2]. Patients with psoriasis often report a substantial negative impact on their daily quality of life, such as a scaly appearance, limitations on clothing, the need to cover up their skin in public places, relationship problems, and fertility problems. The use of less toxic medications and more rapid control of symptoms are the main expectations of patients. For mild-to-moderate psoriasis, medications with serious side effects, such as methotrexate, cyclosporine, or expensive biologics, are not recommended.

Currently, treatment recommendations are based on the severity of psoriasis symptoms: topical preparations (corticosteroids and vitamin D3) for mild-to-moderate psoriasis and systemic or photo-based therapies for moderate-to-severe psoriasis [2]. In China, advances in the understanding of the pathogenesis of psoriasis have led to the development of more efficacious therapeutic options, including oral Chinese medicines, which have been used for thousands of years. The pathogeny of psoriasis is complex and multifactor. The effect of single Chinese medicine will not be obvious, and the compliance of patients will be reduced. Therefore, most of them need a combination therapy. Sugar-free Yinxie granules are a commonly used oral medicine for the treatment of mild-to-moderate psoriasis. Although the use of this medicine alone can produce certain effect, the recommended treatment is in combination with other drugs. Huaier, one of the National First Class New Drugs in China, is a widely available fungus found on the trunks of trees and has been used in traditional Chinese medicine for approximately 1600 years [3]. Huaier not only improves immune regulation but has also recently been established as an antitumor compound. In many tumors, it can effectively inhibit cell proliferation, induce cell-cycle arrest, promote cell apoptosis, and exert a marked antitumor effect [4]. Although the proliferation pattern of psoriatic keratinocytes is a benign hyperplasia, previous studies have shown that many antiproliferative drugs, such as acitretin, methotrexate, and cyclosporine, have been proven effective for the treatment of psoriasis. The purpose of this study was to verify the role of Huaier in the treatment of mild-to-moderate psoriasis in clinical trial, and Hacat cells were used to demonstrate the inhibitory effect of Huaier on the proliferation of epidermal cells.

## 2. Materials and Methods

### 2.1. Clinical Trial

#### 2.1.1. Patients

This clinical study was performed at the Department of Dermatology of the First Affiliated Hospital of Harbin Medical University, between February and July 2017. The study was approved by the local ethics committee of each participating institution. The inclusion criteria for this study were (1) written informed consent voluntarily signed and obtained from the subject; (2) men and women aged between 18 and 64 years; (3) a confirmed diagnosis of mild-to-moderate psoriasis based on the diagnostic criteria listed in the textbook China Clinical Dermatology [4]; (4) BSA affected by psoriasis of ≤ 9%, excluding the scalp, PASI score ≤ 11%; and (5) a suitably engaged patient, able to ensure that the observations throughout the treatment course were valid and the data were accurate. The key exclusion criteria included use of systemic nonbiological psoriasis therapy (methotrexate, cyclosporine, etc.) or psoralen plus UVA/UVB phototherapy in the 4 weeks before screening; treatment with biological agents ≤12 months before the study; breastfeeding women or women planning to become pregnant; or patients with known allergies to HQH or sugar-free Yinxie granules; or a history of severe vital organ disease and other conditions, which the investigators deemed unsuitable for participation in this study. The basic demographics of the two groups of patients are shown in Table 1.

#### 2.1.2. Study Design

The patients were randomly assigned to either the “sugar-free Yinxie granule plus HQH” group (Experiment group A) or “sugar-free Yinxie granule plus placebo” group (Control group B) at a ratio of 1:1. As the pathogenesis of psoriasis is complex and multifactorial, the curing potential of a single traditional Chinese medicine could not be understandable and convincing enough for some of the patients. Given the possibility that this could reduce the compliance, we therefore used a combination therapy in this study. Initially, we planned to enroll approximately 100 patients in each group; 84 and 80 patients completed the study in the experimental and control groups, respectively. The persons responsible for medication management and drug dispensing were blinded for the whole study to achieve objective assessments. For the efficacy evaluation, each score was assessed by the same physician in a blinded fashion before and after treatment. In both treatment groups, sugar-free Yinxie granules (Rongchang Pharmaceutical Co., Ltd. China) were orally administered at 6 g twice per day if the weight of the patient was ≤ 70 kg, or 6 g three times per day if the weight of the patient was > 70 kg. In group A, HQH (Gaitianli Co., Ltd., Ningbo, China), of which the main ingredient is Huaier, was orally administered at 10 g twice per day. In group B, all patients received placebo, which was identical in appearance (HQH-simulating capsule prepared by Gaitianli Medicine Co., Ltd., Ningbo, China) to the active tablet. Both groups were treated for 4 weeks. To aid general skin care, topical Yu Ze body lotion (Shanghai Jahwa, Shanghai, China), a nonmedicated bland emollient, was given to all patients 2 times a day and stopped for 1 day before each visit. This study was conducted in accordance with the Declaration of Helsinki and the Good Clinical Practice guidelines. The institutional review boards at each study site approved the study protocol.

#### 2.1.3. Assessment of Efficacy

The patients were analyzed before treatment (baseline) and at 2 and 4 weeks after the initiation of the treatment. At each follow-up visit, PASI was evaluated and documented. After all subjects completed the 4-week study, the efficacy was evaluated based on the reduction in PASI score from the equation: efficacy index = ([PASI scores before treatment−PASI scores after treatment]/PASI scores before treatment)×100%. The PASI scores were between 0 and 72, with higher scores indicating more severe disease [5]. The percentage of patients achieving 50%, 75%, and 90% reductions in PASI score (PASI 50, PASI 75, and PASI 90, respectively) was compared between the two parallel groups at 2 and 4 weeks after the initiation of the treatment. At weeks 2 and 4, the indicators were assessed, including BSA, sPGA, and DLQI. sPGA was assessed by the investigator, based on their impression of disease severity. A seven-point scale was used to score sPGA, where 0 = clear and 6 = severe.

The primary efficacy endpoints were the percentage improvement in the PASI score from baseline at weeks 2 and 4, the percentage of patients with an sPGA of 0 or 1 at week 4, and the improvement in psoriasis BSA involvement (0%–100%) at week 4. The patient-reported outcomes included the DLQI, which was scored between 0 and 30, and lower scores indicate the disease has a reduced impact on quality of life [6]. The achievement of a DLQI total score of 0 or 1 is important to augment patient satisfaction with the treatment [7]. A comparison of the various scores of two groups after 4 weeks is shown in Table 2.

#### 2.1.4. Safety Assessments

The safety was evaluated for 4 weeks by continuous monitoring of treatment-emergent adverse effects, changes in the laboratory parameters, and changes in vital signs.

### 2.2. Results

**Figure 1:**
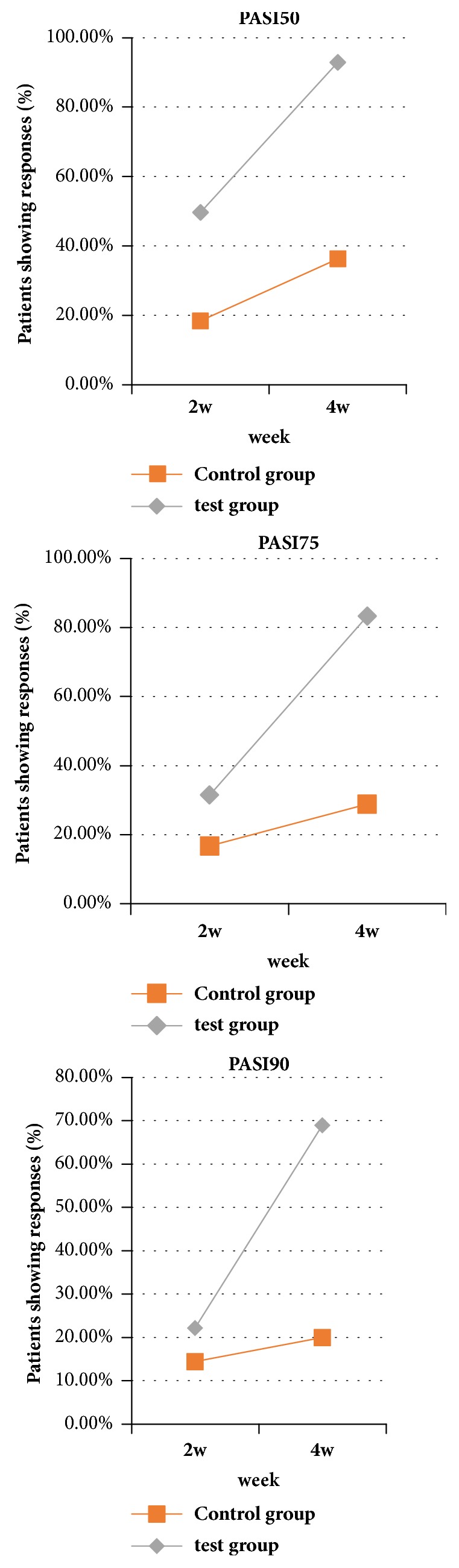
Comparison of PASI between the 2 groups after 2 weeks and 4 weeks of treatment. After 2 weeks of treatment, there was no significant difference in PASI score between the two groups. After treatment for 4 weeks, the number of patients who achieved PASI 50, PASI 75, and PASI 90 was significantly different (P<0.01).

**Figure 2:**
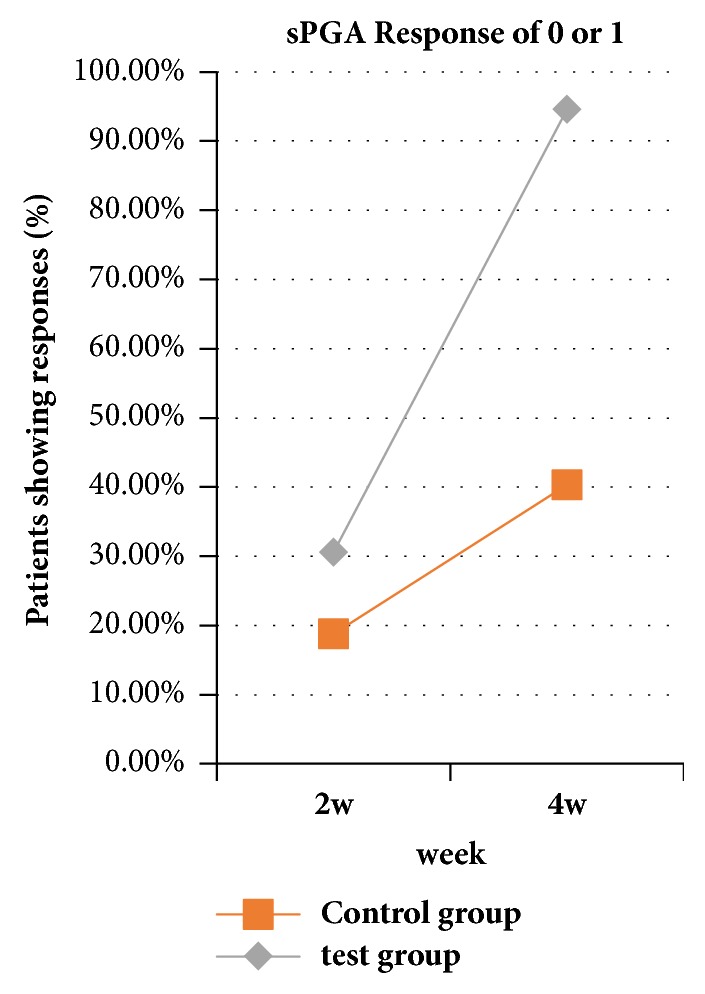
Comparison of sPGA between the 2 groups after 2 weeks and 4 weeks of treatment. After 2 weeks of treatment, there was no significant difference in sPGA score between the two groups. After 4 weeks of treatment, a significant difference was found (P<0.01).

**Figure 3:**
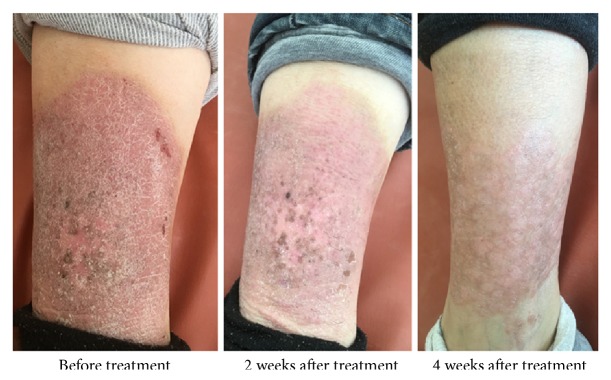
Contrast photos before and after treatment for 41-year-old women in therapy group. The history of disease was 8 months, PASI 8.0, BSA 2.8%, sPGA 3, DLQI 7.0 (before). After 4 weeks of treatment, erythema, desquamation, and infiltration were significantly reduced and basically subsided (PASI 1.8, BSA 2.5%, sPGA 0, DLQI 1.0).

**Figure 4:**
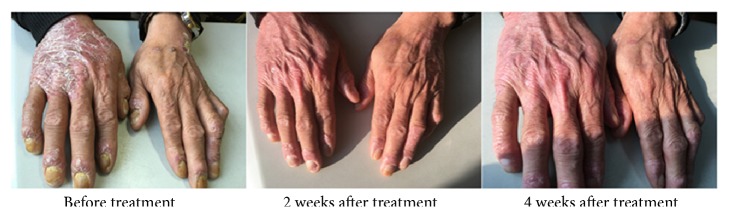
Contrast photos before and after treatment for 60-year-old men in therapy group. The history of disease was 18 months, PASI 8.0, BSA 6.8%, sPGA 3, DLQI 9.0 (before). After 4 weeks of treatment, erythema, desquamation, and infiltration were significantly reduced and basically subsided (PASI 0.5, BSA 0.2%, sPGA 0, DLQI 0).

After treatment for 4 weeks, the number of patients who achieved PASI 50, PASI 75, and PASI 90 was significantly different (P<0.01, Figure 1). The improvements in BSA and sPGA were significantly different (P<0.01, Figure 2), with a significant improvement of DLQI (P < 0.01). In the treatment group, erythema, infiltration, and desquamation of the lesion subsided rapidly. In the 2 weeks after treatment, there was a clear improvement. At 4 weeks, the psoriatic lesions were substantially cleared (Figures 3 and 4).

#### 2.2.1. Adverse Events

The incidence of adverse events was quite low; two cases of diarrhea occurred in the treatment group and one case of bloating occurred in the control group. The above adverse events, which all occurred within the first 3 days of starting treatment, quickly subsided and did not appear again. No other serious adverse reactions or emergencies occurred. Compared with that before treatment, there were no significant differences in blood parameters, liver function, and renal function after treatment by repetitive test.

### 2.3. Cell-Based Experiments

#### 2.3.1. Materials and Methods

*(1) Cell Lines and Reagents*. Hacat cell lines were obtained from American Type Culture Collection (ATCC; Rockville, MD, USA). The electuary ointment of Huaier was kindly provided by Gaitianli Medicine Co., Ltd. (Jiangsu, China) and dissolved in complete medium adequately to obtain the 100 mg/ml stock solution, which was then filtrated with a 0.22 mm filter and stored at 4°C for short-term use. Dulbecco's modified Eagle's medium (DMEM) (GIBCO, USA), fetal bovine serum (FBS) was obtained from ScienCel, USA, and the cell cycle kit was obtained from Beijing 4A Biotech Co., Ltd. (China).

*(2) Cell Culture*. Hacat cells were routinely maintained in DMEM medium supplemented with 10% FBS. The cells were routinely cultured at 37°C in an atmosphere of 5% CO_2_ in a humidified incubator.

*(3) Observation of Hacat Cell Morphology by Optics Microscopy*. Huaier solutions (0, 4, 8, and 16 mg/mL) were used to interact with Hacat cells for 0, 24, 48, and 72 h and fixed with glutaraldehyde. Changes in the morphology and the number of cells were analyzed by optical microscopy (Olympus, Tokyo, Japan).

*(4) CASY Cell Counting for Cell Viability Detection*. A total of 1 × 10^5^ cells/well was seeded in a 6-well culture plate. Huaier was prepared at 2, 4, 8, and 16 mg/mL. The control cells were treated with only DMEM. The plates were incubated 37°C in a humidified atmosphere containing 5% CO_2_. The cells were cultured for 24, 48, and 72 h. CASY-ton buffer (10 mL) buffer was into the CASY cup. A minimum concentration of 2×10^4^ cells/mL was placed in the capillary and these were analyzed by using a CASY Cell Counter and Analyzer System, model TTC (Innovatis, Germany).

*(5) Cell-Cycle Analysis*. Cell-cycle analysis was performed by using the standard method, with some modifications. Briefly, 1.5×10^6^/mL cells/well were seeded in 6-well plates and starved in serum-free medium at 37°C. After starvation for 12 h, the cells were treated with 0, 4, 8, and 16 mg/mL Huaier extract for 48 h, trypsinized, washed with cold phosphate-buffered saline (PBS), and fixed overnight in 75% cold ethanol at -20°C. On the next day, the fixed cells were washed with PBS twice and suspended in 100 *μ*L binding buffer. Staining buffer (500 *μ*L) was added to stain cell DNA in the dark. Propidium iodide staining solution (25 *μ*L) and RNase A (10 *μ*L) were mixed gently in the dark at room temperature for 30 min. Within 1 h, the cells were analyzed in the dark by using a FACScan flow cytometer and the data were analyzed by the ModFitLT V2.0 software (Becton Dickinson).

### 2.4. Results

#### 2.4.1. Huaier-Induced Changes in Morphology and Number of Hacat Cells Measured by Optical Microscopy

**Figure 5:**
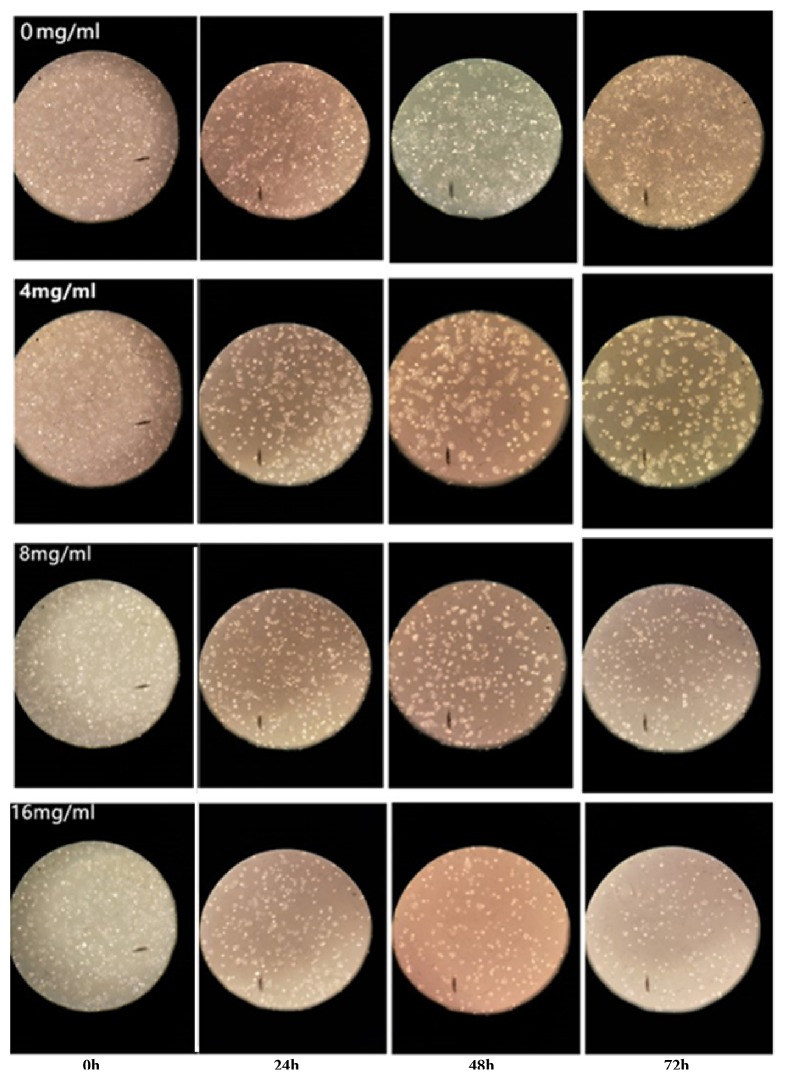
Huaier-induced changes in morphology and number of Hacat cells changes shown by optical microscopy. As the concentration and exposure time increased, the number of Hacat cells gradually decreased, morphological changes occurred, and the cells became irregular.

As the concentration and exposure time increased, the number of Hacat cells gradually decreased; additionally, morphological changes occurred and the cells became irregular (Figure 5).

#### 2.4.2. Huaier-Induced Inhibition of Cell Proliferation and Viability Measured by CASY Assay

**Figure 6:**
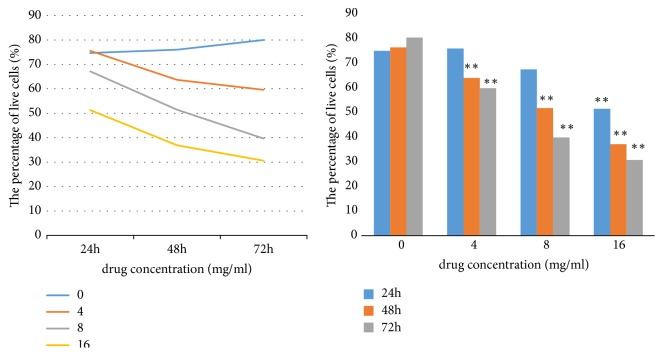
Effect of Huaier on cell viability. CASY cell analysis indicated that Huaier inhibited the viability of Hacat cells in a time- and dose-dependent manner. At 24 h, only the 16 mg/ml group showed significant differences compared with the control group (P < 0.01). At 48 h and 72 h, there was a significant difference in each concentration group compared with the control group (P < 0.01). Increased exposure time and concentration resulted in a decreased cell survival percentage. IC50 (72 h) = 5.78 mg/ml. *∗∗* (P<0.01).

Huaier affected the viability of Hacat cells in a time- and dose-dependent manner; as the exposure time and concentration increased, the cell survival percentage decreased (Figure 6).

#### 2.4.3. Huaier Induces Cell-Cycle Arrest

**Figure 7:**
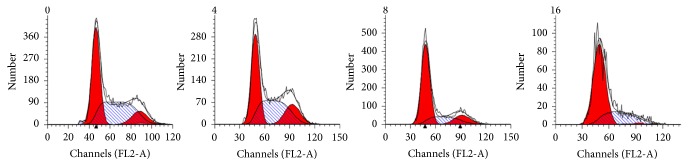
Effect of Huaier on cell-cycle. Huaier induces cell-cycle arrest, blocked Hacat cells in the G1 phase in dose-dependent manner, with significant differences compared with the control group at 4, 8, and 16 mg/mL (P <0.01) after 48 h.

Huaier was able to block the proliferation of Hacat cells in the G1 phase of the cell cycle. This occurred in a dose-dependent manner, with statistically significant differences observed with 4, 8, and 16 mg/mL treatment over 48 h (P <0.01) (Figure 7).

### 2.5. Statistical Analysis

SPSS software (version 17.0) was used to compute the statistical analyses. Student's t-test and one-way ANOVA were performed to determine significance. All error bars were presented as the standard error of the mean (SEM) of three experiments. Differences with a P value of <0.05 were considered statistically significant.

## 3. Discussion

This study presented the first evidence that Huaier affected keratinocyte proliferation and the cell cycle and demonstrated the clinical efficacy and safety of Huaier in subjects with mild-to-moderate psoriasis.

The pathophysiology of psoriasis is diverse, predominantly comprising the hyperproliferation of keratinocytes, blood vessel hyperplasia of dermal papillae, and superficial dermal perivascular inflammation. The keratinocytes of patients with psoriasis were active and the cell cycle was significantly shortened. The epidermal passage time of keratinocytes can be shortened from 28 days to 3–4 days. From these cell-based experiments, it was confirmed that Huaier altered the morphology of Hacat cells, significantly reduced the number of cells, and effectively inhibited the proliferation of Hacat cells by blocking the cell cycle in the G1 phase (P<0.01). CASY cell analysis confirmed that Huaier effectively inhibited the proliferation of Hacat cells in a statistically significant time- and concentration-dependent manner (P <0.01). This is similar to many other studies, which have indicated that Hacat can inhibit the proliferation of tumor cells: previous experiments have confirmed that Huaier effectively inhibited the proliferation of melanoma, breast cancer, colon cancer, liver cancer, prostate cancer, stomach cancer, fibrosarcoma, lung cancer, and many other tumor cells [3, 8–14]; in addition to tumor cells, previous studies have confirmed that Huaier inhibited the proliferation of some nontumor cells, such as mesangial cells and nodular sclerosis cells [15, 16]. This study confirmed that Huaier effectively inhibited the excessive proliferation and accumulation of keratinocytes, which demonstrated an important link to the pathogenesis of psoriasis. This was also thought to be the main reason that the treatment was able to quickly control the symptoms of psoriasis.

Recent studies have confirmed that Huaier is a potent antiangiogenic agent able to inhibit the proliferation of human umbilical vein endothelial cells (HUVECs), reduce HUVEC cell activity and angiogenesis, and reduce microvessel density [17]. In the study of the hepatocellular carcinoma SMMC-7721 tumor-bearing mice, Huaier was found to significantly inhibit hypoxia inducible factor (HIF)-1 protein and vascular endothelial growth factor (VEGF) expression, reduce microvessel density [18], and inhibit macrophage-mediated angiogenesis [19]. These effects of Huaier suggest that it could effectively inhibit microvascular endothelial proliferation and reduce the density of microvessels.

Immune dysfunction also plays an important role in the pathogenesis of psoriasis. Studies on the role of Huaier in the mouse liver cancer (H22) tumor-bearing mice showed that the relative weights of the immune organs (the thymus and spleen) and lymphocyte proliferation were increased after treatment with Huaier [20]. Huaier increased immunostimulatory cytokines, which demonstrated that Huaier enhanced the function of the immune system function [4]. Currently, it is marketed as an immunomodulator.

As Huaier inhibits keratinocyte proliferation and microvascular proliferation, reduces microvessel density, and is a potent immunomodulator, it is expected to exert an antipsoriasis effect. In this clinical trial, the combination of HQH and sugar-free Yinxie granules demonstrated clear advantages in the improvement of PASI score, sPGA score, DLQI score, and BSA score (P<0.01), which confirmed that Huaier is effective in the treatment of psoriasis. In the Huaier treatment group, the patient's psoriatic lesions disappeared more quickly, which was intuitively reflected in the follow-up for each patient. After treatment for 4 weeks, the optimum effect was seen and the quality of life of the patients was significantly improved. The incidence of adverse reactions was low and no serious adverse reactions occurred. The clinical trial supported the results of the cell-based experiments, indicating that Huaier is a potent antipsoriasis drug with few side effects.

These experiments represent a preliminary study into the effects of Huaier on the proliferation of keratinocytes and the evaluation of efficacy and safety of Huaier for the treatment of mild-to-moderate psoriasis. Through cell-based experiments and clinical study, the preliminary results proved the hypothesis. However, there are still some limitations; future experiments are necessary to elucidate other effects of Huaier, such as Hacat cell apoptosis and autophagy. Clinical trials with larger populations are also required to confirm this hypothesis, explore better dosage regimens, and conduct longer-term efficacy observations. The use of Huaier alone is expected to induce various grades of improvement, compared with the placebo, in order to further clarify the role of Huaier in the treatment of psoriasis. The application of primary cultured epidermal cells from patients with psoriasis in cell-based experiments may provide more convincing evidence, but owing to the limitations in cell culture and analysis, this research used Hacat cells; however, the experimental results from primary culture are anticipated.

## 4. Conclusions

Huaier effectively inhibited the proliferation of Hacat cells and induced cell-cycle arrest and the efficacy and safety in patients with psoriasis were observed in clinical trial. Huaier has shown promising effects in both experimental and clinical setting in this preliminary study and it might provide some benefit in the treatment of psoriasis vulgaris in the future.

## Figures and Tables

**Table 1:** The condition of the two groups before treatment.

	Yinxie Granule+HQH (treatment group) n=84	Yinxie Granule+placebo (control group) n=80
Age, years	32.2±10.7	33.4±11.2
Male,%	44	41
Female,%	40	39
Duration of the disease (month)	18.4	17.9
Psoriasis BSA involvement, %	6.90%±0.02%	6.75%±0.03%
sPGA Score, n(%)		
2	43 (51.2)	40 (50.0)
3	39 (46.4)	37 (46.3)
4	2 (2.4)	3 (3.7)
DLQI score	9.4±6.2	9.2±6.9
PASI score	8.28±0.03	8.19±0.05

Data are presented as numbers (%) or the means ± standard deviations.

BSA: body surface area; sPGA: static physician's global assessment; DLQI: Dermatology Life Quality Index; PASI: Psoriasis Area and Severity Index. There was no significant difference between the experimental group and the control group (P>0.05).

**Table 2:** The condition of the two groups after 4 weeks of treatment.

	Yinxie Granule+HQH (treatment group) n=84	Yinxie Granule+placebo (control group) n=80
PASI score	2.27±0.04	6.29±0.03*∗∗*
PASI 50, n (%)	78 (92.9)	29 (36.3)*∗∗*
PASI 75, n (%)	70 (83.3)	23 (28.8)*∗∗*
PASI 90, n (%))	58 (69.0)	16 (20.0)*∗∗*
Improvement in Psoriasis BSA involvement, %	1.61±4.09	5.12±3.50*∗∗*
sPGA clear or almost clear (0 or 1), n (%)	75(89.3)	22 (27.5)^∗∗^
DLQI change from baseline	- 9.1±6.9	-2.0±6.8^∗∗^

Data are presented as numbers (%) or the means ± standard deviations.

*∗∗*p < 0.01, for comparison with control group.

## Data Availability

The data used to support the findings of this study are available from the corresponding author upon request.

## References

[1] Raho G., Koleva D. M., Garattini L., Naldi L. (2012). The burden of moderate to severe psoriasis: An overview. *PharmacoEconomics*.

[2] Kivelevitch D. N., Hebeler K. R., Patel M., Menter A. (2013). Emerging topical treatments for psoriasis. *Expert Opinion on Emerging Drugs*.

[3] Zhang F., Zhang Z., Liu Z. (2013). Effects of Huaier aqueous extract on proliferation and apoptosis in the melanoma cell line A875. *Acta Histochemica*.

[4] Song X., Li Y., Zhang H., Yang Q. (2015). The anticancer effect of Huaier. *Oncology Reports*.

[5] Zhao B. (2009). *China Clinical Dermatology*.

[6] Feldman S. R., Krueger G. G. (2005). Psoriasis assessment tools in clinical trials. *Annals of the Rheumatic Diseases*.

[7] Finlay A. Y., Khan G. K. (1994). Dermatology Life Quality Index (DLQI)—a simple practical measure for routine clinical use. *Clinical and Experimental Dermatology*.

[8] Gao S., Li X., Ding X., Jiang L., Yang Q. (2017). Huaier extract restrains the proliferative potential of endocrine-resistant breast cancer cells through increased ATM by suppressing miR-203. *Scientific Reports*.

[9] Sun W., Dou J., Zhang L. (2017). Killing effects of Huaier Granule combined with DC-CIK on nude mice transplanted with colon carcinoma cell line. *Oncotarget*.

[10] Hu Z., Yang A., Su G. (2016). Huaier restrains proliferative and invasive potential of human hepatoma SKHEP-1 cells partially through decreased Lamin B1 and elevated NOV. *Scientific Reports*.

[11] Yang A.-L., Hu Z.-D., Tu P.-F. (2016). Study on effect and mechanism of Huaier aqueous extract on growth and invasion of human prostate cancer PCL cells. *Zhongguo Zhongyao Zazhi*.

[12] Xie H., Xu Z. Y., Tang J. N. (2015). Effect of Huaier on the proliferation and apoptosis of human gastric cancer cells through modulation of the PI3K/AKT signaling pathway. *Experimental and Therapeutic Medicine*.

[13] Cui Y., Meng H., Liu W., Wang H., Liu Q. (2015). Huaier aqueous extract induces apoptosis of human fibrosarcoma HT1080 cells through the mitochondrial pathway. *Oncology Letters*.

[14] Wu T., Chen W., Liu S. (2014). Huaier suppresses proliferation and induces apoptosis in human pulmonary cancer cells via upregulation of miR-26b-5p. *FEBS Letters*.

[15] Bai J., Geng W., Mei Y. (2017). Effect of Huaier On the Proliferation of Mesangial Cells in Anti-Thy-1 Nephritis. *Cellular Physiology and Biochemistry*.

[16] Yang A., Fan H., Zhao Y. (2016). Huaier aqueous extract inhibits proliferation and metastasis of tuberous sclerosis complex cell models through downregulation of JAK2/STAT3 and MAPK signaling pathways. *Oncology Reports*.

[17] Wang X., Zhang N., Huo Q., Yang Q. (2012). Anti-angiogenic and antitumor activities of Huaier aqueous extract. *Oncology Reports*.

[18] Li C., Wu X., Zhang H. (2015). A Huaier polysaccharide restrains hepatocellular carcinoma growth and metastasis by suppression angiogenesis. *International Journal of Biological Macromolecules*.

[19] Li Y., Qi W., Song X., Lv S., Zhang H., Yang Q. (2016). Huaier extract suppresses breast cancer via regulating tumor-associated macrophages. *Scientific Reports*.

[20] Li C., Wu X., Zhang H. (2015). A Huaier polysaccharide inhibits hepatocellular carcinoma growth and metastasis. *Tumor Biology*.

